# Identification of influential observations in high-dimensional cancer survival data through the rank product test

**DOI:** 10.1186/s13040-018-0162-z

**Published:** 2018-02-14

**Authors:** Eunice Carrasquinha, André Veríssimo, Marta B. Lopes, Susana Vinga

**Affiliations:** IDMEC, Instituto Superior Técnico, Universidade de Lisboa, Rovisco Pais, 1, Lisbon, Portugal

**Keywords:** Survival analysis, Data dimensionality reduction, Rank product test, Gene expression

## Abstract

**Background:**

Survival analysis is a statistical technique widely used in many fields of science, in particular in the medical area, and which studies the time until an event of interest occurs. Outlier detection in this context has gained great importance due to the fact that the identification of long or short-term survivors may lead to the detection of new prognostic factors. However, the results obtained using different outlier detection methods and residuals are seldom the same and are strongly dependent of the specific Cox proportional hazards model selected. In particular, when the inherent data have a high number of covariates, dimensionality reduction becomes a key challenge, usually addressed through regularized optimization, e.g. using Lasso, Ridge or Elastic Net regression. In the case of transcriptomics studies, this is an ubiquitous problem, since each observation has a very high number of associated covariates (genes).

**Results:**

In order to solve this issue, we propose to use the Rank Product test, a non-parametric technique, as a method to identify discrepant observations independently of the selection method and deviance considered. An example based on the The Cancer Genome Atlas (TCGA) ovarian cancer dataset is presented, where the covariates are patients’ gene expressions. Three sub-models were considered, and, for each one, different outliers were obtained. Additionally, a resampling strategy was conducted to demonstrate the methods’ consistency and robustness. The Rank Product worked as a consensus method to identify observations that can be influential under survival models, thus potential outliers in the high-dimensional space.

**Conclusions:**

The proposed technique allows us to combine the different results obtained by each sub-model and find which observations are systematically ranked as putative outliers to be explored further from a clinical point of view.

## Background

One of the statistical techniques most used in the medical field is survival analysis, whose goal is to study the time until an event of interest and its associated covariates. The event may be death, the relapse of a tumour, or the development of a disease. The response variable is the time until that event, called survival or event time, which can be censored, i.e. not observed on all individuals present in the study.

In this context, the Cox proportional hazards regression model [1] is the classical approach to deal with this type of censored data. It is based on a semi-parametric likelihood since the baseline hazard function, *h*_0_(*t*), is not specified, which contributes to its flexibility. Although the Cox regression model is a widely used method due to its simplicity, the corresponding estimator has a breakdown point of 1/*n* [2], which means that the presence of outlying observations may have extreme influence on the estimation of the model parameters. In order to handle this problem, a robust version of the Cox regression model has also been proposed [3].

The robust version of the Cox regression model [3] is based on doubly weighting the partial likelihood function of the Cox regression model. The robust Cox is an alternative method to the Cox regression model estimation, as a framework that allows to infer the parameters in a more robust way when outlying observations are present, i.e., individuals that lived too long or died too early when compared to others with the same clinical conditions. Furthermore, the weights obtained with this method can give information about which observations are more influential and therefore can be considered as putative outliers [4].

The detection of outliers in survival data has gained great importance due to the fact that the identification of individuals with survival time too high or too short can lead in the medical field to the detection of new prognostic factors [5]. The first attempts to analyze and to identify outliers were based on residuals. In this context, graphical methods based on the analysis of martingale, score and deviance residuals were proposed [6], and also other contributions including the log-odds and normal deviate residuals [5].

One of the challenges arising when dealing with patient’ omics data is the high-dimensionality problem. In this type of data, the number of covariates (*p*) is often much larger than the number of observations (*n*), i.e., *p*≫*n*. In this context, the usual statistical techniques for the estimation of the parameters cannot be applied, due to the inherent ill-posed inverse problem [7].

When dealing with thousands of covariates, as is the case for omics data, dimensionality reduction is a crucial initial step, leading to distinct models depending on the variable selection method used.

In this context, regularized optimization techniques are widely applied, which include the *least absolute shrinkage and selection operator* (Lasso) [8], Ridge and Elastic Net regularization [9]. The Lasso, uses an *l*_1_-norm regularizer, and the Elastic Net uses a linear combination of *l*_1_ and *l*_2_ penalties. In contrast with the Elastic net, in the presence of highly correlated variables, the Lasso tends to arbitrarily select one of them.

In this sense, depending on the methodology used to reduce the dimensionality of the data, different models are obtained and, consequently, distinct outliers are identified. The aim of this work is, therefore, given a high-dimensional dataset, to find outliers (or influential observations) from different sub-models, which are obtained from distinct techniques for variable selection. The method proposed is based on the Rank Product (RP) test, a non-parametric method, to identify the outliers that are consistently highly ranked in each of the sub-models. The ovarian cancer dataset, with gene expressions as covariates, was chosen to illustrate the applicability of the proposed method. Three gene expression sub-models are presented, and the RP test is applied as a consensus or ensemble test that combines the results obtained by each model, often distinct and sometimes contradictory. Notice that each sub-model has different baselines, since for this particular dataset there is no groundtruth to start from.

Although the rank product and the deviances measures for survival models were already proposed previously in different contexts, the combination of RP-based statistical tests as a means of conferring robustness to outlier detection tasks represents the main novelty of this work.

The outline of this work is as follows. In “Methods” section, the martingale residual used to detect outliers in survival analysis and the Rank Product test are explained in detail. In “Results” section the results concerning an application example are presented. Finally, Conclusions are addressed in “Conclusions” section.

## Methods

The method proposed to obtain potential outliers considering different sub-models, is the Rank Product (RP) test. Before explaining this technique in detail, we need to select the measure used to obtain outliers in survival analysis.

There are in the literature a vast number of ways to identify abnormal (outlying) observations in survival analysis. The most common technique is based on the residuals, as referred before. More recent studies proposed other algorithms based on quantile regression [10] and the concordance c-index [11]. In the present work the focus will be given to the martingale residual but it is worth mentioning that the proposed method can be applied to any other deviance measures, as long as a final outlyingness ranking can be obtained.

The Martingale residuals arise from a linear transform of the Cox-Snell residuals [6] and are very useful for outlier detection for censored data.

Let all the covariates be fixed, the martingale residual for the *i*^*th*^ individual is given by

1 $$ \hat{r}_{{M}_{i}}=\delta_{i}-\hat{H}_{0}(t_{i})\exp\left(\hat{\boldsymbol{\beta}}^{T}\mathbf{x}_{i}\right),   $$

where ***β***=(*β*_1_,…,*β*_*p*_) are the unknown regression coefficients, which represent the covariate effect in the survival, $\hat {H}_{0}(t_{i})$ represents the estimate of the cumulative baseline hazard, **x**_*i*_=(*x*_*i*1_,…,*x*_*ip*_) is the covariate vector associated with the *i*^*th*^ individual and *δ*_*i*_ is the censored function. These residuals are asymmetric and take values in (−*∞*,1).

The martingale residuals are the difference between the observed number of the events for the *i*^*th*^ individual in (0,*t*_*i*_) and the corresponding expected number, obtained by the adjusted model. The observed number of ‘deaths’ is one if *t*_*i*_ is not censored, i.e., is equal to *δ*_*i*_. On the other hand, *r*_*i*_ is the estimate of *H*(*t*_*i*_), which can be interpret as the expected number of ‘deaths’ in (0,*t*_*i*_), since it is only considered an individual.

This residuals will reveal the individuals that are not well adjusted to the model. i.e., those that lived too long (large negative values) or died too soon (values near one), when compared to other individuals with the same covariate pattern.

### Rank product (RP)

When dealing with high dimensional datasets, dimensionality reduction is warranted. Regularization methods are an example on how to overcome this challenge, as referred to before. However, different technique result in different estimated sub-models, which will significantly influence the obtained results regarding the identification of outlying cases.

In order to address this challenge, we propose a method that can combine all the results obtained for each one of the different sub-models. The rationale is that, if a given observation is systematically classified as an outlier, independently of the chosen sub-model, then our trust on the accuracy of that particular classification should increase. To accomplish this goal, the RP test is used.

From the theoretical point of view, the RP test is a non-parametric statistical technique which gained great importance in detecting deferentially regulated genes in replicated microarray experiments [12] and allowing the meta-analysis of independent studies [13].

The required input is a list of all the observations ranked by their level of outlyingness, based on one of the described methods for outlier detection. The backbone of this method is to allow the statistical assessment of a consensus rankings obtained in distinct sub-models, thus providing a combined identification of observations consistently ranked higher.

From the conceptual point of view, let *n* be the number of observations and *k* the number of different sub-models where the outlier detection method was performed. Consider that *Z*_*ij*_ is a measure of the deviance (or outlyingness) of the *i*^*th*^ observation in the *j*^*th*^ sub-model, with 1≤*i*≤*n* and 1≤*j*≤*k*. The deviance rank for each *Z*_*ij*_ considering method *j* is defined by

2 $$ R_{ij}=rank(Z_{ij}), \qquad 1\leq R_{ij} \leq n.   $$

For each sub-model, the lowest ranks imply that the observation is more outlier that the others. After obtaining the ranks for each sub-model, the rank product is performed,

3 $$ RP_{i}=\prod_{j=1}^{k}R_{ij}.   $$

Several methods were proposed in order to estimate the statistical significance of *RP*_*i*_ under the null hypothesis of random (uniform) rankings. In [12] the distribution of *RP*_*i*_ was based on a permutation approach. An alternative formulation that is less computational intensive was described more recently, based on an approximation of the logarithm of those values using the gamma distribution with parameters (*k*,1) [14]. In [15] the exact probability distribution for the rank product was derived. The one chosen in the present study is based on the geometric mean of upper and lower bounds, defined recursively [16], since the algorithm provides accurate approximate *p*-values for the rank product when compared to the exact ones and is substantially faster in terms of computational execution.

Another key issue when performing these tests is related with the multiple testing problem. In fact, since many observations are tested, type-I errors (false positives) will increase. Several correction methods exist that usually adjust *α* so that the probability of observing at least one significant result due to chance remains below a desired significance level. The Bonferroni correction is one classical choice, with less conservative options also available, such as the False Discovery Rate (FDR) [17].

The FDR, which is the expected proportion of false positives among all tests that are significant, sorts in an ascendant order the *p*-values and divides them by their percentile rank. The measure used to determine the FDR is the *q*-value. For the *p*-value: 0.05 implies that 5% of all tests will result in false positives, instead, for the *q*-value: 0.05 implies that 5% of significant tests will result in false positives. The *q*-value is therefore able to control the number of false discoveries in those tests. For this reason it has the ability of finding truly significant results.

In this context, the RP is used as a consensus technique for all different results obtained by each sub-model. In order to illustrate this approach, the RP technique is applied to three sub-models, where the goal is to obtain outlying observations based on the martingale residuals, independently of the estimated sub-model. In order to evaluate the dependency of the results to the particular choice of the sub-models, a resampling strategy was also conducted.

## Results

To evaluate the proposed consensus outlier detection method, the described procedure was applied to a high-dimensional dataset constituted by ovarian cancer patients microarray expression data.

This dataset was obtained from The Cancer Genome Atlas (TCGA) (http://cancergenome.nih.gov/) and is constituted by 517 observations (patients) over 12,042 covariates, comprising follow-up times, survival status and microarray gene expressions of all the patients (https://gdc-portal.nci.nih.gov/).

For the analysis, this dataset was aggregated by the TCGA consortium allowing for the analysis to be reproducible with the original dataset. The clinical data was cleaned using “Days to last follow-up” and “Days to death” attributes to detect inconsistencies between them. Only the cases where the number of days matched were included in the analysis. The same process was performed for the attributes “Days to death” and “Vital status”, where some cases had as status “deceased”, but a missing “Days to death”.

This dataset was analyzed in three different ways. In the first analysis the following regularization methods were performed [18]: 1) Lasso, 2) Lasso and elastic net, leading to two different sets of selected genes. The union of these sets was then considered, allowing to reduce the dimensionality from 12,042 to 109 covariates (genes). After this, a stepwise algorithm using the AIC (Akaike information criterion) was applied and 63 covariates were thus obtained. In the second analysis, 18 genes were considered, based on those selected in a previous study [19]. Finally, a third approach is presented where 22 genes were selected based on their reported association with ovarian cancer, as in the Genetics Home Reference https://ghr.nlm.nih.gov/condition/ovarian-cancer%23genes. The list included also gene *RAD51D* which is not present in the original TCGA data and was therefore discarded from the analysis. Notice that for the three analysis considered there is no overlap of the covariates selected.

It is noteworthy that, although we have pursued these three analyses, we can indeed include many others, for example, using different feature selection methods or prior clinical information.

To overcome the fact that the results obtained for each of the analysis are model-based, a sampling strategy was also implemented in order to determine whether resampling the data using a sub-model of covariates (genes) would recognize the outliers previously identified. The resampling algorithm randomly picked 1000 genes (without replacement) from the ovarian cancer dataset. The Cox regression model with elastic-net regularization was then fitted (using glmnet), resulting in a reduced set of selected genes. In order to calculate the corresponding martingale residuals, a Cox regression is then performed on this reduced gene set (using coxph). The resulting residuals allow to sort the observations accordingly to their outlyingness level. This procedure is repeated 100 times, resulting in 100 models to feed the RP test.

All the analysis were performed in R [20] and are fully documented in the “Rmd File” as R Mardown files to allow full reproducibility. The libraries used for the analysis were: survival, for the Cox regression model to obtain the martingale residuals, and qvalue, to determine the *q*-values. The two robust versions of the Cox regression model were the coxrobust, and an improvement of this method available in [4]. The algorithm implementation to obtain the *p*-values for the rank product, based on the geometric mean, is provided by Heskes and colleagues [16].

The proportional hazard assumption [21] for the Cox’s regression model was tested, and the results showed that this hypothesis was never violated. The *p*-values for each of the sub-model presented are the following: 0.1932 (63 genes), 0.3795 (18 genes) and 0.3868 (22 genes).

The majority of gene expression do not have a normal distribution (see Supplementary files for the Shapiro tests conducted) although this fact does not affect the resulting Cox models’ validity.

In the next sections the results for the martingale residual, for each one of the models, and the RP that combine all the ranks, for each sub-model considered, is presented.

### Outlier detection results for each sub-model

#### TCGA ovarian cancer - 63 genes

**Fig. 1:**
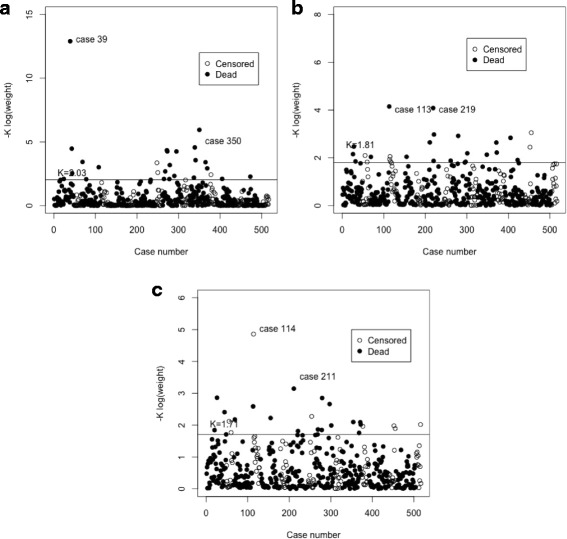
Plot of robust estimates with log-transformed exponential weight vs. case number for the TCGA ovarian cancer data for each one of the sub-models. **a** 63 genes expression, **b** 18 genes expression, **c** 22 genes expression

**Fig. 2:**
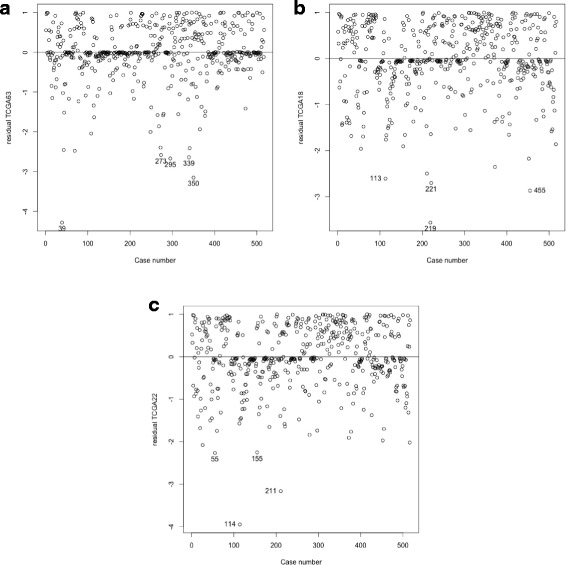
Plot of the martingale residuals for the TCGA ovarian cancer data for each one of the sub-models. **a** 63 genes expression, **b** 18 genes expression, **c** 22 genes expression

For this particular model, the dataset can be represented as a matrix of size 517×63. The Cox’s regression and the Cox’s robust regression models were performed. The following 21 genes were significant for a 5% level of significance in all the methods considered: *HPCA*, *RPS6KA2*, *GRB7*, *ABCD2*, *WDR76*, *NDUFA3*, *PI3*, *BNC1*, *D4S234E*, *CSNK1G1*, *SSTR1*, *PSG3*, *GAS1*, *POPDC2*, *DAP*, *SRY*, *HOXD11*, *HSPA1L*, *PPP3CA*, *MPZ* and *LBP*. Also 11 genes for the Cox proportional hazard and 13 genes for the Cox robust, were not significant, for a 5% level of significance. Those differences are regarding genes: *SDF2L1*, *PRR16*, *ALG8* and *ELA3A*. Genes *SDF2L1*, *PRR16* and *ALG8* were not significant in the Cox robust and significant in the Cox regression model and gene *ELA3A* was significant in the robust case ([4], proposal) and not significant for the classical Cox. For more details, see Table 1. Figure 1 shows that observations 39 and 350 are identified as influential observations in the sense that they have the lowest weights. The results regarding the residuals are shown in Fig. 2. Again observations 39 and 350 in the martingale residuals appears to have the lowest values when compared to all the others.

**Table 1:** Results for the Cox’s regression model and Cox’s robust (both proposals) for the TCGA data with 63 genes

	Cox			CoxRobust ([3])			CoxRobust ([4])		
Genes	coef	se(coef)	*p*-value	coef	se(coef)	*p*-value	estimate	SE	*p*-value
HPCA	-1.1893	0.3560	0.0008	-1.1803	0.5877	0.0446	-1.1662	0.3387	0.0006
UBE2J1	-0.2160	0.1475	0.1431	-0.2221	0.2676	0.4064	-0.2220	0.1364	0.1035
RPS6KA2	0.2972	0.1124	0.0082	0.3892	0.1408	0.0057	0.3980	0.1201	0.0009
SDF2L1	-0.2025	0.1024	0.0480	-0.2003	0.1203	0.0959	-0.1979	0.1017	0.0516
GRB7	0.3360	0.0965	0.0005	0.3268	0.1115	0.0034	0.3272	0.0873	0.0002
PTGFR	1.1771	0.4891	0.0161	1.0255	0.6001	0.0875	1.0131	0.4899	0.0386
ABCD2	2.1329	0.7532	0.0046	2.3397	1.1928	0.0498	2.3564	0.7860	0.0027
FLJ20323	0.2936	0.1322	0.0264	0.2696	0.1480	0.0685	0.2654	0.1251	0.0338
WDR76	1.1471	0.3040	0.0002	1.1701	0.5071	0.0210	1.1695	0.3387	0.0006
NDUFA3	0.3454	0.1352	0.0106	0.4128	0.1633	0.0115	0.4130	0.1289	0.0014
FJX1	-0.1945	0.0987	0.0488	-0.2867	0.1616	0.0760	-0.2934	0.1023	0.0041
GAPDHS	0.8798	0.5092	0.0840	0.9733	0.6198	0.1163	0.9929	0.5517	0.0719
RAB40B	-0.1852	0.0833	0.0263	-0.2219	0.1404	0.1140	-0.2232	0.0838	0.0077
PRR16	-0.4071	0.1887	0.0310	-0.3362	0.2740	0.2198	-0.3367	0.1863	0.0707
CLTCL1	0.3730	0.2601	0.1515	0.4470	0.3452	0.1953	0.4354	0.2817	0.1223
PPM2C	0.3999	0.1005	0.0001	0.4173	0.2192	0.0569	0.4160	0.1027	0.0001
FOXE3	-0.8118	0.5080	0.1100	-0.5162	0.6139	0.4005	-0.5129	0.4706	0.2757
CHIT1	-0.9427	0.2741	0.0006	-0.9042	0.4674	0.0531	-0.9102	0.3584	0.0111
PI3	0.2450	0.0466	0.0000	0.2305	0.1083	0.0333	0.2310	0.0443	0.0000
BNC1	0.1648	0.0693	0.0174	0.1830	0.0847	0.0307	0.1837	0.0731	0.0120
D4S234E	-0.1471	0.0606	0.0153	-0.1645	0.0767	0.0319	-0.1664	0.0636	0.0089
SAPS2	0.8055	0.2158	0.0002	0.8342	0.6100	0.1714	0.8345	0.2133	0.0001
CSNK1G1	0.8805	0.3858	0.0225	1.0782	0.4489	0.0163	1.0874	0.3901	0.0053
MLL2	1.0106	0.4972	0.0421	1.3137	0.8978	0.1434	1.3255	0.5169	0.0103
HSPB7	0.6657	0.3540	0.0600	0.5092	0.4368	0.2437	0.5004	0.3526	0.1559
SLC37A4	-0.2538	0.1635	0.1205	-0.3065	0.2269	0.1768	-0.3142	0.1653	0.0573
WTAP	0.5562	0.1590	0.0005	0.5607	0.3265	0.0860	0.5599	0.1563	0.0003
SSTR1	-1.7443	0.6359	0.0061	-1.7979	0.7908	0.0230	-1.8039	0.6710	0.0072
IDUA	1.4248	0.4480	0.0015	1.4354	0.8810	0.1032	1.4447	0.4714	0.0022
PSG3	-2.1008	0.7371	0.0044	-2.3029	0.8579	0.0073	-2.2998	0.7673	0.0027
SLC9A2	0.3374	0.1267	0.0077	0.3185	0.1677	0.0575	0.3179	0.1311	0.0153
PAPOLG	1.8006	0.4837	0.0002	1.7430	0.9548	0.0679	1.7445	0.4623	0.0002
GAS1	0.2589	0.0861	0.0027	0.2756	0.1380	0.0458	0.2785	0.0854	0.0011
ELA3A	-0.4516	0.2360	0.0557	-0.4692	1.1530	0.6840	-0.4715	0.2266	0.0375
KIF26B	0.9000	0.2329	0.0001	0.8508	0.4996	0.0886	0.8502	0.2299	0.0002
GBP2	-0.3527	0.0935	0.0002	-0.3718	0.1924	0.0532	-0.3749	0.0959	0.0001
POPDC2	-3.0285	0.4894	0.0000	-2.7792	1.2267	0.0235	-2.7675	0.5214	0.0000
OPN1SW	2.3693	0.5099	0.0000	2.1049	1.0821	0.0518	2.1140	0.5087	0.0000
DAP	-0.7017	0.1333	0.0000	-0.6959	0.2120	0.0010	-0.6957	0.1307	0.0000
SRY	-2.3810	0.7835	0.0024	-2.4342	1.0015	0.0151	-2.4382	0.7497	0.0011
UTP20	0.3955	0.1553	0.0109	0.4170	0.2133	0.0506	0.4185	0.1589	0.0084
HOXD11	0.8313	0.2268	0.0003	0.7056	0.2897	0.0149	0.7047	0.2147	0.0010
HSPA1L	0.3765	0.1828	0.0395	0.4634	0.2344	0.0480	0.4645	0.2207	0.0353
PPP3CA	0.3213	0.1113	0.0039	0.3294	0.1262	0.0091	0.3316	0.1019	0.0011
PAX2	-0.2296	0.0899	0.0106	-0.2373	0.2193	0.2792	-0.2375	0.0869	0.0063
FZD10	-0.0994	0.0553	0.0720	-0.0801	0.0748	0.2841	-0.0807	0.0563	0.1518
TREML2	-0.6339	0.4228	0.1339	-0.6043	0.5415	0.2644	-0.6143	0.4665	0.1879
CCR7	-0.6175	0.2637	0.0192	-0.5713	0.4291	0.1830	-0.5692	0.2349	0.0154
MPZ	0.8243	0.2329	0.0004	0.7611	0.3173	0.0164	0.7626	0.2097	0.0003
MGAT4C	1.1627	0.6331	0.0663	1.0216	0.6915	0.1396	1.0177	0.5374	0.0583
EHMT1	1.8125	0.4705	0.0001	1.5360	1.0943	0.1604	1.5220	0.4978	0.0022
ALG8	-0.2209	0.1067	0.0385	-0.1276	0.1482	0.3894	-0.1188	0.1135	0.2950
KCNN2	-1.1298	0.3040	0.0002	-1.1903	1.0630	0.2628	-1.1909	0.2916	0.0000
ESR2	-2.6987	1.0408	0.0095	-2.4160	1.7091	0.1575	-2.4447	1.1388	0.0318
TGM2	-0.2265	0.1370	0.0982	-0.1904	0.2393	0.4262	-0.1907	0.1667	0.2526
LBP	1.0330	0.2216	0.0000	0.9934	0.2712	0.0002	0.9919	0.2492	0.0001
SRPK3	-0.7770	0.2074	0.0002	-0.8033	0.4268	0.0599	-0.8068	0.1927	0.0000
FBXO40	1.4431	0.5331	0.0068	1.3587	0.7145	0.0572	1.3517	0.5519	0.0143
ANGPT2	-0.3112	0.1571	0.0477	-0.3140	0.1849	0.0894	-0.3151	0.1393	0.0237
IRF5	-0.8805	0.3143	0.0051	-0.8175	0.5146	0.1121	-0.8176	0.3097	0.0083
ANXA4	0.2854	0.1191	0.0166	0.2839	0.1674	0.0900	0.2852	0.1350	0.0346
DENND2D	-0.2540	0.1053	0.0159	-0.2419	0.1388	0.0813	-0.2416	0.0957	0.0116
SGEF	-1.4599	0.6064	0.0161	-1.4272	0.8081	0.0774	-1.4264	0.6434	0.0266

#### TCGA ovarian cancer - 18 genes

The features selected were based on the work of [19] where the authors considered as covariates of the Cox model the expression of 18 genes. The dataset is a matrix of size 517×18, and, in this case, the only genes statistically significant were: *CRYAB* and *SPARC*, for Cox’s and Cox’s robust (see Table 2).

**Table 2:** Results for the Cox’s regression model and Cox’s robust (both proposals) for the TCGA data with 18 genes

	Cox			CoxRobust ([3])			CoxRobust ([4])		
Genes	coef	se(coef)	*p*-value	coef	se(coef)	*p*-value	estimate	SE	*p*-value
*LPL*	0.1263	0.0751	0.0924	0.1011	0.0856	0.2375	0.1011	0.0717	0.1584
*IGF1*	0.0210	0.0600	0.7266	0.0341	0.0705	0.6289	0.0340	0.0670	0.6114
*EDNRA*	0.0224	0.1227	0.8549	0.0619	0.2119	0.7704	0.0621	0.1482	0.6752
*MFAP5*	0.0165	0.0482	0.7327	0.0089	0.0622	0.8865	0.0089	0.0516	0.8630
*LOX*	0.1918	0.1251	0.1254	0.1688	0.1499	0.2604	0.1690	0.1281	0.1872
*INHBA*	-0.1432	0.1786	0.4227	-0.1556	0.1895	0.4118	-0.1556	0.1841	0.3978
*THBS2*	0.0639	0.0902	0.4787	0.0863	0.1072	0.4205	0.0862	0.0908	0.3422
*ADIPOQ*	-0.1256	0.0910	0.1676	-0.0727	0.1047	0.4875	-0.0728	0.1001	0.4667
*NPY*	0.0552	0.0496	0.2655	0.0625	0.0710	0.3785	0.0625	0.0553	0.2590
*CCL11*	-0.1296	0.0960	0.1771	-0.1578	0.1212	0.1927	-0.1576	0.1013	0.1197
*VCAN*	0.0578	0.1009	0.5664	0.0286	0.1419	0.8404	0.0286	0.0956	0.7651
*DCN*	0.0729	0.0892	0.4133	0.0791	0.0993	0.4257	0.0791	0.0976	0.4176
*TIMP3*	0.0719	0.0835	0.3891	0.0775	0.0906	0.3925	0.0775	0.0881	0.3789
***CRYAB***	0.1092	0.0424	0.0100	0.1179	0.0544	0.0302	0.1180	0.0437	0.0069
*CXCL12*	0.0204	0.0818	0.8030	0.0129	0.0962	0.8932	0.0130	0.0879	0.8826
***SPARC***	-0.3811	0.1402	0.0066	-0.3978	0.2020	0.0489	-0.3975	0.1332	0.0029
*CNN1*	0.0863	0.1141	0.4493	0.1313	0.1395	0.3468	0.1313	0.1341	0.3275
*FBN1*	0.1135	0.1690	0.5018	0.1122	0.2234	0.6154	0.1116	0.1806	0.5365

Highlighted in bold are statistically significant genes, in this case *CRYAB* and *SPARC*

The *CRYAB* gene codes for the crystallin alpha B chain, a protein that acts as a molecular chaperone. Its function is to bind misfolded proteins and, interestingly, some defects associated to this protein and gene have already been associated with cancer, among other diseases. In particular, a recent study [22] analyzed which molecular factors could affect ovarian cancer cell apoptosis and the authors found out that there was a statistical significant association between the expression of crystallin B (C*CRYAB*) with survival. This protein has, indeed, a negative regulation of tumor necrosis, which may explain these results.

The *SPARC* gene codes for Secreted protein acidic and rich in cysteine, a protein that appears to be a regulator of cell growth, by interaction with cytokines, the extracellular matrix and also binding calcium, copper, and several others biochemical compounds. This protein is overexpressed in ovarian cancer tissues [23], playing a central role in growth, apoptosis and metastasis. It also has been identified as a candidate therapeutic target [24].

Figure 1 shows that observations 113 and 219 are identified as influential observations (lowest weights). However, for this example, the weights are not so distinct in the sample. The results regarding the residuals are shown in Fig. 2. Observation 219 in the martingale residuals has the lowest value when compared to the all the others.

#### TCGA ovarian cancer - 22 genes

The dataset considered is a matrix of size 517×22, where the number of columns corresponds to the genes that are most associated with the ovarian cancer. Interestingly, only two genes in this dataset are statistically significant: *BRCA2* in the Cox model, and *PALB2* when considering both Cox model and its Heritier’s robust version [4] (see Table 3).

**Table 3:** Results for the Cox’s regression model and Cox’s robust (both proposals) for the TCGA data with 22 genes

	Cox			CoxRobust ([3])			CoxRobust ([4])		
Genes	coef	se(coef)	*p*-value	coef	se(coef)	*p*-value	estimate	SE	*p*-value
*AKT1*	-0.1991	0.1028	0.0526	-0.1793	0.1714	0.2954	-0.1794	0.1054	0.0888
*BARD1*	-0.0363	0.1145	0.7512	-0.0471	0.1227	0.7010	-0.0473	0.1118	0.6724
*BRCA1*	0.0984	0.1595	0.5375	0.1467	0.2017	0.4669	0.1462	0.1657	0.3776
***BRCA2***	0.4940	0.2114	0.0194	0.4092	0.2403	0.0886	0.4093	0.2195	0.0623
*BRIP1*	-0.2211	0.2395	0.3558	-0.1447	0.2869	0.6141	-0.1446	0.2541	0.5694
*CDH1*	0.0377	0.1422	0.7908	-0.0133	0.1903	0.9441	-0.0135	0.1790	0.9400
*CHEK2*	-0.1278	0.1007	0.2045	-0.0877	0.1118	0.4325	-0.0875	0.1043	0.4012
*CTNNB1*	0.1986	0.1702	0.2433	0.1555	0.2419	0.5204	0.1554	0.1673	0.3530
*MLH1*	0.0662	0.1443	0.6464	0.0004	0.1541	0.9981	0.0004	0.1530	0.9977
*MRE11A*	-0.1625	0.2097	0.4385	-0.2578	0.3052	0.3983	-0.2577	0.2133	0.2270
*MSH2*	0.0412	0.1340	0.7588	0.1081	0.2364	0.6475	0.1083	0.1331	0.4159
*MSH6*	0.0441	0.2101	0.8339	-0.0298	0.3432	0.9309	-0.0298	0.1953	0.8789
*NBN*	0.1908	0.1149	0.0967	0.1790	0.1530	0.2420	0.1790	0.1256	0.1542
*OPCML*	0.3367	0.3194	0.2919	0.3620	0.3162	0.2522	0.3616	0.2366	0.1264
***PALB2***	-0.4238	0.1385	0.0022	-0.3886	0.2140	0.0694	-0.3884	0.1522	0.0107
*PARK2*	0.7468	0.5007	0.1358	0.6960	0.6044	0.2495	0.6957	0.5059	0.1690
*PIK3CA*	0.0086	0.1012	0.9326	0.0426	0.1171	0.7157	0.0427	0.1067	0.6893
*PMS2*	0.1267	0.1210	0.2951	0.1077	0.1561	0.4901	0.1078	0.1265	0.3940
*RAD50*	0.1426	0.1317	0.2789	0.1794	0.1527	0.2402	0.1792	0.1439	0.2129
*RAD51C*	-0.0955	0.1163	0.4114	-0.0844	0.1383	0.5418	-0.0844	0.1210	0.4857
*STK11*	0.0616	0.3449	0.8582	0.1420	0.3867	0.7134	0.1422	0.3641	0.6960
*TP53*	-0.0485	0.0624	0.4371	-0.0521	0.0908	0.5659	-0.0520	0.0665	0.4339

Highlighted in bold are statistically significant genes, in this case *BRCA2* and *PALB2*

Both *BRCA2* and *PALB2* genes encodes a protein that may function in tumor suppression (for more details see https://ghr.nlm.nih.gov/gene/. In the BRCA2 this protein is to help repair damaged DNA ensuring the stability of the cell’s genetic material. If the *BRCA2* gene is mutated/changed the DNA could be corrupted developing genetic alterations that can lead to cancer. In [25] is conducted a study where *BRCA1* and *BRCA2* genes mutations account for the majority of hereditary ovarian carcinomas. On the other hand the *PALB2* is related to breast cancer. Recent studies [26] showed that women who carry mutations in the *PALB2* gene are at similar breast cancer risks as those who carry mutations in *BRCA2*.

When using the weights of the robust version, 114 is identified as an influential observation (Fig. 1). Figure 2 shows the results concerning the residuals. Observations 114 and 211 in the martingale residuals have the lowest values when compared to all the others.

To overcome the fact that, for each sub-model, different outliers are obtained, the RP test was performed. The results are presented in the next section.

### Rank Product results

The ranks of the martingale residuals for each sub-model were determined. The product of the ranks was obtained, and, finally, the *p*-values and corresponding *q*-values were calculated, as shown in Table 4. Based on those results, and considering a 5% level of significance, the observations that are considered outliers based on the three different sub-models are: 55, 114, 211, 219 and 455.

**Table 4:** Ranks for outlier detection using the martingale residual sorted by *q*-value, for each sub-model

ID	Time	Status	Rank Martingale	Rank Martingale	Rank Martingale	*p*-value	*q*-value
			18 genes	22 genes	63 genes		
114	2780	0	11	1	25	4.31E-05	0.0223
55	2967	0	8	3	29	1.39E-04	0.0324
211	3953	0	5	2	90	1.88E-04	0.0324
219	3525	0	1	32	54	3.96E-04	0.0496
455	3532	0	2	13	79	4.79E-04	0.0496
115	2259	0	14	21	14	1.02E-03	0.0752
279	2688	1	21	9	19	8.80E-04	0.0752
377	2078	0	38	10	15	1.43E-03	0.0824
452	5481	0	7	7	113	1.39E-03	0.0824
155	2982	0	9	4	232	2.13E-03	0.0916
221	2788	0	3	16	188	2.30E-03	0.0916
372	3096	0	6	8	155	1.89E-03	0.0916
516	3825	0	10	6	147	2.25E-03	0.0916
26	3622	1	35	5	58	2.59E-03	0.0958
69	2490	1	73	29	6	3.25E-03	0.1120

Notice that three of the observations considered as outliers in the RP test had low values for the martingale residual. Observation 219 for the model with 18 genes, and observations 114 and 211 for the model that considered 22 genes.

The overall values of the survival time are between 8 to 5481 days, with the first, second and third quantile: 376, 923 and 1483, respectively. Only approximately 3% of the observations had a survival time higher than 2500 days. Regarding observations 114, 211 and 219 the survival time is, respectively, 2780, 3953 and 3525 (maximum was 5481 days), all censored, see Table 4. In this way the observations identified are long-term survivors.

To illustrate the robustness of the RP test, a resampling technique was performed as described above. The results displayed in Table 5 show that the observations considered outliers for the three different sub-models are also outlying observations for the 100 different models obtained. This includes all the observations considered outliers in Table 4. Indeed, there are individuals that consistently appear with larger residuals, irrespectively of the model. It is noteworthy that, although the genes selected in each model are different, there is a set of patients that always exhibit discrepant values for their survivals, as would be predicted by their covariates. This illustrates the robustness of the method to a particular choice of the model.

**Table 5:** Top 25 of the outliers obtained for the resampling technique for 100 models, selecting 1000 genes sorted by *q*-value

ID	Rank Mart. 1	Rank Mart. 2	Rank Mart. 3	Rank Mart. 4	Rank Mart. 5	…	Rank Mart. 96	Rank Mart. 97	Rank Mart.	Rank Mart. 99	Rank Mart. 100	*p*-values	*q*-values
372	35	40	5	8	2	…	62	10	19	10	90	≈0	≈0
314	26	6	8	2	31	…	12	22	1	8	14	≈0	≈0
377	19	2	1	22	16	…	1	21	32	18	5	≈0	≈0
348	103	5	72	33	14	…	36	1	6	12	13	≈0	≈0
452	18	45	9	10	7	…	24	9	2	15	19	≈0	≈0
219	2	16	18	16	5	…	120	33	5	3	22	≈0	≈0
39	4	4	69	1	1	…	17	102	136	17	3	≈0	≈0
115	41	27	37	38	34	…	33	14	9	32	25	≈0	≈0
113	15	146	6	24	104	…	104	152	3	41	57	≈0	≈0
338	178	19	38	68	63	…	27	56	29	74	236	≈0	≈0
516	29	44	12	67	64	…	3	3	10	5	30	≈0	≈0
211	28	12	15	11	117	…	29	15	58	11	75	≈0	≈0
55	51	46	24	9	12	…	35	8	13	29	8	≈0	≈0
455	9	13	17	34	40	…	26	18	8	116	114	≈0	≈0
301	52	35	3	12	103	…	18	47	50	1	12	≈0	≈0
220	5	9	13	23	28	…	10	19	15	16	34	≈0	≈0
11	37	28	7	13	33	…	16	44	60	44	50	≈0	≈0
350	1	37	41	120	80	…	5	167	103	7	27	≈0	≈0
69	32	38	36	37	13	…	21	12	61	14	39	≈0	≈0
32	22	26	22	47	75	…	51	50	7	21	32	≈0	≈0
114	31	20	19	55	50	…	22	27	11	20	20	≈0	≈0
44	97	15	78	17	60	…	71	4	178	13	86	≈0	≈0
210	61	81	46	40	41	…	11	46	30	26	42	≈0	≈0
117	23	61	33	15	19	…	13	90	42	62	71	≈0	≈0
119	87	51	27	58	32	…	47	89	33	50	65	≈0	≈0

These results show that the proposed method was able to combine in a statistically solid way the results of different estimated models. In particular, the application of the RP test allowed to identify a consensual list of putative outliers in the dataset in a semi-automatic way, paving the way for the analysis of other datasets where discrepant observations are a critical issue in clinical applications.

## Conclusions

The aim of this work was to propose a combined method based on the RP test. The proposed technique allows to combine the different results obtained by each sub-model and find which observations are systematically ranked as putative outliers. By the application examples tested, it can be seen that the results regarding outlier detection are highly dependent on the specific method used. In fact for a certain dataset the choice of the covariates used significantly changes the outliers identified, which may hamper a definite answer in this respect. Therefore, the results regarding the influential observations in a given dataset are highly depended on the specific model adjusted. The proposed application of the RP test nevertheless illustrates that it is possible to combine the different results and to obtain a consensus list of putative outliers to be explored further from a clinical point of view.

## Abbreviations

FDR: False discovery rate
RP: Rank product
